# Transplantation of a heart donated after circulatory death via thoraco-abdominal normothermic regional perfusion and results from the first Spanish case

**DOI:** 10.1186/s13019-020-01372-z

**Published:** 2020-11-25

**Authors:** Marina Pérez Redondo, Sara Alcántara Carmona, Susana Villar García, Alberto Forteza Gil, Héctor Villanueva Fernández, Francisco José Hernández-Pérez, José Luis Campo-Cañaveral de la Cruz, Rocío Velasco Calvo, Javier Segovia-Cubero, Beatriz Alonso Menárguez, Francisco del Río Gallegos, Elisabeth Coll, Beatriz Domínguez-Gil González, Juan José Rubio Muñoz

**Affiliations:** 1 Department of Intensive Care Medicine Department of Donor and Transplant Coordination, Hospital Universitario Puerta de Hierro Majadahonda. Instituto de investigación sanitaria Puerta de Hierro – Segovia de Arana, Madrid, Spain; 2 Department of Intensive Care Medicine, Hospital Universitario Puerta de Hierro Majadahonda, Madrid, Spain; 3 Department of Cardiac Surgery, Hospital Universitario Puerta de Hierro Majadahonda, Madrid, Spain; 4 Department of Cardiology. Advanced Heart Failure and Transplant Unit, Hospital Universitario Puerta de Hierro Majadahonda, Madrid, Spain; 5 Thoracic Surgery and Lung Transplantation Department, Hospital Universitario Puerta de Hierro-Majadahonda, Madrid, Spain; 6 Department of Neurology, Hospital Universitario Puerta de Hierro de Majadahonda, Madrid, Spain; 7 CIBER cardiovascular, Instituto de Salud Carlos III, Madrid, Spain; 8 Division of Transplant and Cardiac Anesthesia, Department of Anesthesiology, Hospital Universitario Puerta de Hierro Majadahonda, Madrid, Spain; 9 Regional Transplant Coordinator Community of Madrid, Madrid, Spain; 10 Organización Nacional de Trasplantes, Madrid, Spain

**Keywords:** Controlled donation after circulatory death, Heart transplantation, Normothermic regional perfusion, Case report

## Abstract

**Background:**

Controlled donation after circulatory death (cDCD) has emerged as one of the main strategies for increasing the organ donor pool. Because of the ischemic injury that follows the withdrawal of life-sustaining therapies, hearts from cDCD donors have not been considered for transplantation until recently. The ex-situ perfusion of hearts directly procured from cDCD donors has been used to allow the continuous perfusion of the organ and the assessment of myocardial viability prior to transplantation. Based on our experience with abdominal normothermic regional perfusion in cDCD, we designed a protocol to recover and validate hearts from cDCD donors using thoraco-abdominal normothermic regional perfusion without the utilization of an ex-situ device.

**Case presentation:**

We describe the first case of a cDCD heart transplant performed with this approach in Spain. The donor was a 43-year-old asthmatic female diagnosed with severe hypoxic encephalopathy. She was considered a potential cDCD donor and a suitable candidate for multiorgan procurement including the heart via thoraco-abdominal normothermic regional perfusion. The heart recipient was a 60-year-old male diagnosed with amyloid cardiomyopathy. Cold ischemia time was 55 min. The surgery was uneventful.

**Conclusions:**

This case report, the first of its kind in Spain, supports the feasibility of evaluating and successfully transplanting cDCD hearts without the need for ex-situ perfusion based on the use of thoraco-abdominal normothermic regional perfusion opening the way for multiorgan donation in cDCD.

## Background

Controlled donation after circulatory death (cDCD) refers to the donation from individuals who have been declared dead following the decision for the withdrawal of life-sustaining therapies (WLST) that are no longer considered beneficial to patients with devastating brain injury or terminal lung, heart and neurodegenerative diseases [1]. cDCD is one of the most robust strategies for increasing the organ donor pool and is now carried out in a growing number of countries throughout the world [2]. cDCD was implemented in Spain approximately 10 years ago. Since then, this type of donation has increased exponentially to the point where, nowadays, it accounts for 32% of all deceased donation procedures in the country [3]. Amongst cDCD donors, the use of abdominal normothermic regional perfusion (A-NRP) based on the use of extracorporeal membrane oxygenation (ECMO) devices has become a common practice in several European countries mainly due to the favorable results obtained in liver grafts and the lower rate of delayed graft dysfunction observed in kidney recipients [4–8]. In Spain, A-NRP was used in nearly 50 % of all cDCD cases in 2019.

Initially, cDCD donors were not considered eligible heart donors. It was assumed that the hypoxia suffered by the heart following WLST and during cardiac arrest would deem the organ unsuitable for transplantation due to the irreversible myocardial injury, but different publications in children and adults have shown otherwise [9–11]. The use of either direct procurement followed by ex-situ Organ Care System (OCS™) or thoraco-abdominal normothermic regional perfusion (TA-NRP) with ECMO also followed in the majority of cases by OCS™, allows for the restoration of cardiac function and heart evaluation after the determination of death and prior to transplantation with successful post-transplant outcomes [10–14].

**Fig. 1:**
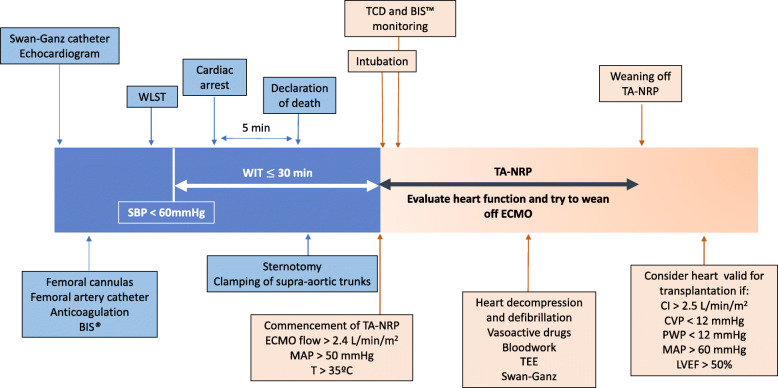
Protocol for cardiac procurement with thoraco-abdominal NRP after cDCD. BIS™: Bispectral Index. WLST: withdrawal of life sustaining therapies. SBP: systolic blood pressure. WIT: warm ischemia time. TA-NRP: thoraco-abdominal normothermic regional perfusion. ECMO: extracorporeal membrane oxygenation. MAP: mean arterial pressure. TCD: transcranial Doppler. TEE: transesophageal echocardiogram. CI: cardiac index. CVP: central venous pressure. PWP: pulmonary wedge pressure. LVEF: left ventricular ejection fraction

Based on these previous experiences and our own experience with the use of A-NRP in cDCD, the Donor Transplant Coordination Unit of Puerta de Hierro Majadahonda University Hospital (Madrid, Spain), together with the Departments of Cardiology, Cardiac Surgery, Thoracic Surgery and Intensive Care, designed a protocol for the procurement of cDCD donor hearts (Fig. 1), based on the use of TA-NRP with no ex-situ preservation devices. The protocol was approved by the Institutional Review Board, the *Organización Nacional de Trasplantes* [National Transplant Organization] and the Transplant Committee of the National Healthcare System at the end of 2019. The protocol was shared with all donor hospitals in the region, with the request to refer any potential cDCD donor who met the eligibility criteria for heart donation. We report the first case of a heart transplantation from a cDCD donor in Spain using this approach.

## Case presentation

A 43-year-old female, with no previous medical history except asthma, suffered a prolonged cardiac arrest secondary to respiratory distress due to a *status asthmaticus*. After the return of spontaneous circulation, following advanced cardio-pulmonary resuscitation, she was admitted to the Intensive Care Unit (ICU) where she developed persistent myoclonus. A magnetic resonance imaging scan of the brain and an electroencephalogram were performed in the following days, and she was diagnosed with severe hypoxic encephalopathy. After 16 days, her neurological situation had not improved, and the decision was made with the patient’s family for WLST. Once this decision had been made, the option of organ donation was presented to the family, who agreed to proceed after considering that organ donation was consistent with the patient’s principles and values. The hospital’s Donor Coordinator considered the potential cDCD donor as a suitable candidate for multiorgan procurement including the heart. Because heart recovery was contemplated, our hospital was contacted, and the potential donor was transferred to our center with authorization from the donor’s family after specific and detailed information about the cDCD procedure had been given to them.

Once the patient was admitted to our ICU, a Swan-Ganz catheter was inserted through the right jugular vein and a transthoracic echocardiogram was obtained as part of the heart evaluation. After the heart and the abdominal organs were evaluated and deemed suitable for transplantation, she was transferred to the operating room where two femoral cannulas, artery and vein, were inserted using a Seldinger’s technique. This technique was also used to place a catheter in the contralateral femoral artery that would be used during TA-NRP to measure arterial pressures. During these procedures, analgesia and sedation were adjusted according to the donor’s needs. Once the cannulas and the femoral artery catheter were in place, the donor was anticoagulated using unfractionated heparin as previously reported [15]. Brain activity was monitored using the Bispectral Index (BIS™) in order to guarantee adequate levels of sedation during the WLST [16].

Once the surgical field was prepared, the family was brought into the operating room. WLST and end-of-life care was conducted following the hospital protocol and performed by the intensive care physician in charge of the patient’s care. Cardiac arrest was diagnosed by the absence of a pulse wave in the femoral artery, and death was declared after a five-minute no-touch period.

Following the declaration of death, a sternotomy was performed, the pericardium was opened, the supra-aortic trunks were clamped and TA-NRP was started with an ECMO flow of 3 L/min/m^2^, aiming for a mean arterial pressure > 50 mmHg and a T > 35 °C. At that point, a norepinephrine infusion was started, reaching a maximum dose of 0.1 mcg/kg/min during organ procurement. Simultaneously, the donor was intubated, and mechanical ventilation was started using a FiO_2_ of 1, a PEEP of 5 and a tidal volume of 6–8 ml per kg of predicted body weight. We consider the warm ischemia time (WIT) as the time from significant hypoperfusion, defined by a systolic blood pressure < 60 mmHg, until TA-NRP is started. A WIT < 30 min for the heart and liver and < 60 for the kidneys is considered valid [15]. The WIT for our case was 16 min.

**Fig. 2:**
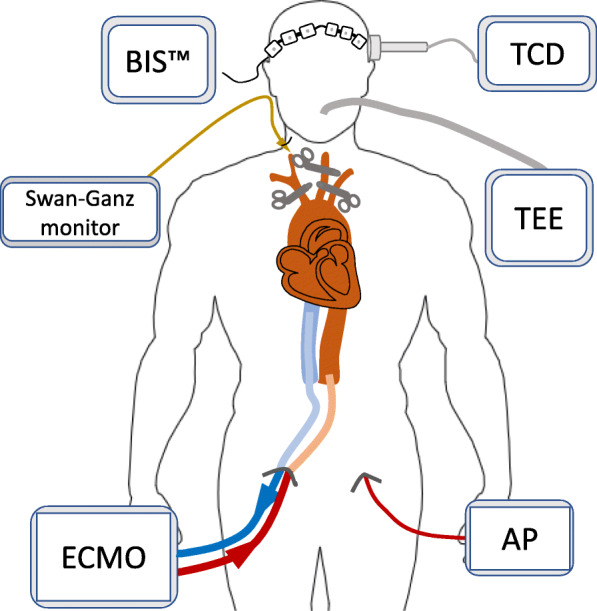
Donor monitoring during thoraco-abdominal NRP. BIS™: Bispectral Index. TCD: transcranial doppler. TEE: transesophageal echocardiogram. AP: femoral arterial pressure. ECMO: extracorporeal membrane oxygenator

One minute after the start of the TA-NRP, a spontaneous effective heartbeat was observed with a normal sinus rhythm. Cardiac output was measured using both the Swan-Ganz catheter and a transesophageal echocardiogram, which also addressed cardiac contractility. The absence of cerebral blood flow was confirmed by transcranial Doppler performed both in the anterior and posterior territories. Brain activity was monitored using the BIS™ which showed values of 00 and a suppression rate of 100 throughout the TA-NRP procedure (Fig. 2). Blood samples to determine arterial blood gas, lactate, troponin I, hematocrit, hemoglobin and liver function parameters were collected every 30 min during TA-NRP (Table 1).

**Table 1:** Data collected before the withdrawal of life-sustaining therapies (baseline) and during thoraco-abdominal normothermic regional perfusion

	Baseline	TA-NRP				
		1′	15′	30′	60′	90′
ECMO flow (L/min)	0	3	0	1	1	1
Cardiac index (L/min/m^2^)^a^	3.9	3.7	3.2	3.7	3.7	3.7
LVEF (%)	74		54			
SpO_2_ (%)	97	100	89	100	100	100
p_a_O_2_ (mmHg)	91.8	423	62	486	455	383
p_a_CO_2_ (mmHg)	48.8	55.5	57.2	25.8	22.8	24.0
pH	7.37	7.09	7.16	7.44	7.49	7.46
HCO_3_^−^ (mEq/L)	28.4	17.2	20.5	17.8	17.7	17.3
Lactate	0.7	8.4	4.8	4.2	3.8	3.6
Hematocrit (%)	34.5	38.4	29.3	29.7	29.9	26.6
Hemoglobin (g/dL)	11.3	12.6	9.5	9.7	9.8	8.7
Troponin I (μg/dl)	0.13	1.91	1.51	1.91	2.18	2.12
ALT (IU/L)	21	21		20	93	138
AST (IU/L)	24	35		33	130	181
Bilirubin (mg/dL)	0.5	0.4		0.3	0.5	0.4

*LVEF* Left Ventricular Ejection Fraction measured by echocardiography, *TA-NRP* Thoraco-Abdominal Normothermic Regional Perfusion

^a^Calculated by Swan-Ganz monitoring

Once heart function was assessed, the ECMO flow was brought down until it reached 0 L/min/m^2^ 15 min after its commencement. Because the donor had a history of severe asthma, SpO_2_ and P_a_O_2_ worsened after TA-NRP was stopped. For this reason, we decided not to wean the donor off ECMO completely, and a flow of 1 L/min/m^2^ was maintained during organ recovery. The duration of organ procurement was 120 min, and the heart, the liver and the kidneys were recovered and transplanted.

The heart recipient was a 60-year-old male diagnosed with amyloid cardiomyopathy. Due to the novelty of the case, the recipient had previously been informed of the peculiarities of the transplant which he agreed to by signing an specific informed consent were the different aspects of the procedure were detailed. Cold ischemia time was 55 min. The surgery was uneventful. He was easily weaned off cardiopulmonary bypass, and effective heartbeat was achieved after one defibrillation. He was then admitted to the ICU on low dose norepinephrine (< 0.2 mcg/kg/min) and low dose dobutamine (< 5 mcg/kg/min) and was extubated after 36 h. Follow-up echocardiograms showed a normal biventricular function. Five months after the transplant he has resumed a normal life.

## Discussion and conclusion

Donor shortage worldwide has led to the development of different strategies to increase the organ donor pool. Amongst these strategies, cDCD has emerged as one of the cornerstones for this growth. It is estimated, according to different data, that the utilization of hearts from cDCD donors would have the potential of increasing the heart transplant activity between 15 to 50% depending on the countries [17, 18]. In Spain, based on a raw analysis that includes cDCD donors under 45 years of age, and excludes those with a suspicion of cardiac injury, it is estimated that the inclusion of these donors would account for a 5–10% increase in the number of heart donors, which would increase the number of hearts transplanted by 15–30 hearts per year.

In order to optimize heart function, one of the inclusion criteria of our protocol was that cDCD heart donors had to be under 45 years of age, a fact that narrowed the number of potential donors. For this reason, it was necessary to build a collaborative approach with other donor hospitals. Given the complexity of the recovery procedure in the case of a cDCD heart, together with the relatively small size of the Autonomous Community of Madrid (8.022 km^2^), we considered that the best approach would be to transfer the potential cDCD heart donor to our own hospital. The previous implementation of an intensive care program to facilitate organ donation in our country has made this a routine practice, whereas small donor hospitals cannot take on sophisticated cDCD procedures [19].

In Spain, ante-mortem interventions aimed at organ preservation that do not interfere with the dying process are not forbidden before WLST as long as the family consents [20]. Ante-mortem cannulation helps to reduce the duration of WIT. The possibility of restoring circulation to the brain – which would retroactively negate the diagnosis of death based on circulatory criteria - is one concern associated to the use of NRP. In the case of A-NRP, it is necessary to inflate an aortic occlusion balloon or to surgically clamp the abdominal aorta before A-NRP starts. In TA-NRP, the ECMO flow cannot start before the supra-aortic trunks have been clamped in order to avoid cerebral perfusion. This maneuver may prolong WIT but, in our case, the sternotomy-to-clamping time was only 4 min, resulting in a WIT of 16 min, which is below the 30-min limit that had been previously stipulated.

The absence of anterior and posterior cerebral perfusion during the procurement, one of the key points of this novel approach, was demonstrated by transcranial doppler, and cerebral electrical activity was monitored with BIS™ which always remained in the 00 mark and showed a suppression rate of 100 indicating an isoelectric electroencephalogram [13, 16, 21, 22].

At present, the majority of hearts recovered from cDCD donors have undergone a period of ex-situ perfusion in an OCS™ [10–12]. However, two recent reports have revealed the feasibility of evaluating and successfully transplanting cDCD hearts without the need for ex-situ perfusion based on the use of TA-NRP [13, 14]. In our case, due to our previous experience with A-NRP, we favored this approach [15]. We also consider that one of the advantages of in-situ heart evaluation is that it allows for a more realistic assessment of the heart through well validated techniques like Swan-Ganz or echocardiography. Also, because this was a case of a multiorgan donor, it was essential to guarantee adequate liver and kidney perfusion during their evaluation and procurement. For this reason, liver function tests were monitored every 30 min. Lactate levels were also measured as both markers of organ perfusion and liver function. Although no cut-off values of lactate were established, a decreasing trend was used as a surrogate for appropriate organ perfusion and adequate liver function during AT-NRP [8]. In all, the recovery procedure took around 120 min. In our case, the lungs were not considered due to the donor’s previous history of severe asthma.

Because both the procurement and the transplant were done in the same center, the cold ischemia time was only 55 min. In the future, it will be necessary to address if hearts procured by this technique will tolerate longer cold ischemia times in order to be implanted at a center different from where they have been retrieved. For the time being, our protocol only contemplates procurement and implantation at the same center in order to minimize the duration of cold ischemia, which may lead to better results in the recipient.

Overall, this is one of the few cases in the world, and the first in Spain, of a cDCD heart retrieved using only TA-NRP and successfully implanted, and it opens up the way for multiorgan donation in cDCD. Given the high cost of ex-situ machine perfusion, unaffordable in many settings, TA-NRP may become an option to make heart transplantation from cDCD donors economically feasible for some countries.

## Data Availability

Being a case report, data sharing not applicable to this article as no datasets were generated or analysed during the current study.

## Abbreviations

A-NRP: Abdominal normothermic regional perfusion
AP: Arterial pressure
BIS™: Bispectral index
cDCD: Controlled donation after circulatory death
CI: Cardiac index
CVP: Central venous pressure
ECMO: Extracorporeal membrane oxygenation
ICU: Intensive Care Unit
LVEF: Left ventricular ejection fraction
MAP: Mean arterial pressure
OCS™: Organ care system
PWP: Pulmonary wedge pressure
SAP: Systolic arterial pressure
TA-NRP: Thoraco-abdominal normothermic regional perfusion
TCD: Transcranial doppler
TEE: Transesophageal echocardiogram
WIT: Warm Ischemia time
WLST: Withdrawal of life-sustaining therapies
